# An evaluation of community pharmacy-based services for type 2 diabetes in an Indonesian setting: patient survey

**DOI:** 10.7717/peerj.1449

**Published:** 2015-12-10

**Authors:** Yosi Wibowo, Richard Parsons, Bruce Sunderland, Jeffery Hughes

**Affiliations:** 1 Centre for Medicines Information and Pharmaceutical Care (CMIPC), Faculty of Pharmacy, University of Surabaya, Surabaya, East Java, Indonesia; 2 School of Pharmacy, Faculty of Health Sciences, Curtin University, Perth, Western Australia, Australia

**Keywords:** Pharmacy services, Indonesia, Community pharmacy, Diabetes, Patient views

## Abstract

**Background.** Diabetes is an emerging chronic disease in developing countries. Its management in developing countries is mainly hospital/clinic based. The increasing diabetes burden in developing countries provides opportunities for community pharmacists to deliver a range of services. Since the management of diabetes requires the patient’s own involvement, it is important to gain their views in order to develop pharmacy-based diabetes services. Studies on diabetes patients’ views have been limited to developed countries.

**Objectives.** To investigate, within a developing country setting (Indonesia), current use of pharmacy services by type 2 diabetes patients, and to evaluate their views regarding community pharmacists’ roles, and the characteristics that influence their views.

**Methods.** A questionnaire survey was conducted within 10 purposefully selected community pharmacies in Surabaya, Indonesia. Each pharmacy recruited approximately 20 patients seeking antidiabetic medications. Usage of pharmacy services was identified using binary responses (‘yes’/‘no’) and views on pharmacists’ roles were rated using Likert scales; an open-ended question was used to identify patient perceived priority roles. Logistic regression models were used to determine characteristics associated with patients’ views.

**Results.** A total of 196 pharmacy patients with type 2 diabetes responded (58.3% response rate). Most patients used community pharmacies for dispensing (100%) and education on how to use medications (79.6%). There were mixed views towards pharmacists providing services beyond dispensing. The highest priorities identified were from the ‘patient education’ domain: education on medications (i.e., directions for use (64.5%), storage (26.6%), common/important adverse effects (25.5%)); and the ‘monitoring’ domain: monitoring medication compliance (37.3%). Patients with higher incomes or who were working were less supportive of these expanded services, whereas patients who previously used a service, those with risk factors for complications or having poor/unknown glycaemic control were more supportive.

**Conclusions.** Community pharmacies in Surabaya, Indonesia in this study were mainly utilised for dispensing. However, many type 2 diabetes patients using these pharmacies report limited monitoring of blood glucose levels and poor glycaemic control, which indicates an opportunity for greater pharmacist involvement. Yet for this to occur, patients’ limited expectations of pharmacists roles will need to be broadened. Characteristics influencing these views should inform the development of pharmacy-based diabetes services in the environment of the burgeoning burden of diabetes.

## Introduction

Indonesia is a major developing country with a population of 237.6 million (International Diabetes Federation , 2013), and is among the top 10 countries in the world according to the number of people with diabetes (Fowler, 2008). In 2013, it was estimated that 8.5 million people in Indonesia were living with diabetes, and this number is expected to increase to 14.1 million by 2035 (Fowler, 2008). Diabetes in Indonesia is currently managed in hospital outpatient or clinic settings (Soewondo et al., 2010; Soewondo, Ferrario & Tahapary, 2013). An increased number of people with diabetes will require more community-based care, providing an opportunity for community pharmacists to deliver a range of diabetes services. While the 2012 Indonesia Health Profile reported a total of 17,613 pharmacies (mainly community-based) (Kementrian Kesehatan Republik Indonesia, 2013), Indonesian community pharmacies currently have limited roles within Government insurance plans, providing services mainly to the private sector (Wang et al., 2009).

The introduction of Standards for Pharmaceutical Care in Community Pharmacies in 2006 has emphasised the need for community pharmacists to be involved in the care of patients with chronic diseases, including diabetes (Kementrian Kesehatan Republik Indonesia, 2006). The standards included a range of services, i.e., prescription medication service (i.e., prescription review, drug dispensing/supply, drug information and counselling, and monitoring), health promotion and education to promote self-care, and home/residential care (Kementrian Kesehatan Republik Indonesia, 2006). A previous study involving a survey of community pharmacists in Surabaya, Indonesia reported despite community pharmacists’ expressed willingness to take up a broader role in diabetes care, the majority performed limited services beyond dispensing (Wibowo et al., 2015). Studies in developed countries (such as the UK, the USA, Canada and European countries), however, reported that more than 50% of community pharmacies have provided extended services for diabetes patients, including: education related to medications, lifestyle education, supporting patients in performing self-monitoring of blood glucose (SMBG), and monitoring compliance with medications (Simpson et al., 2009; Douglas, Power & Hudson, 2007; Abduelkarem et al., 2003b; Younis, Campbell & Slack, 2001; Kjome, Sandberg & Granås, 2008; Plake, Chesnut & Odorzynski, 2007; Storimans, 2005; Timmer et al., 1999).

To increase the uptake of pharmacy-based services amongst diabetes patients in Indonesia, it is important to understand the perspectives of diabetes patients on pharmacy-based services that would assist with their care. This is especially so since diabetes is a chronic disease that requires daily care in the hands of patients (Abduelkarem et al., 2003a). Several studies have been conducted to investigate diabetes patient views regarding aspects of community pharmacists’ roles; however, these have thus far been limited to developed countries, such as the UK and the USA (Abduelkarem et al., 2003a; Weitzman et al., 2009; Brown & Green, 2000; Hermansen-Kobulnicky & Worley, 2008; Twigg et al., 2013). Two previous small studies conducted in community pharmacies in Indonesia, although not specific to diabetes patients, have found that general patients had positive perceptions of pharmacy services, providing facilitation for pharmacists to develop their professional roles (Handayani & Raharni, Gitawati, 2009; Abdullah, Andrajati & Supardi, 2010). This present study aimed to investigate, within an Indonesian setting, the current use of community pharmacy services by patients with type 2 diabetes, and to evaluate their views on the potential roles of community pharmacists, and the characteristics that influence their views. The results of the study should inform the Government, professional bodies, and practitioners on the development of pharmacy-based diabetes services in a developing country setting (Indonesia).

## Methods

This study was approved by the Human Research Ethics Committee of Curtin University (PH-09-11) and *Ikatan Apoteker Indonesia*—IAI (Indonesian Pharmacists Association) (001/SK/BPD-IAI/SURABAYA/2010).

### Setting and sample recruitment

The aims of the study included the estimation of the prevalence of service usage and patient views. A sample size of 200 was defined which if broadly representative of the population of pharmacy patients with type 2 diabetes in Surabaya, the 95% confidence intervals for the true prevalence estimates would be within 7% of the figures obtained from the sample (based on a prevalence estimate of 50%). For the analysis of characteristics associated with respondents’ views, a sample of 200 would be expected to be adequate to identify any independent variables exhibiting a moderate to small effect size (with power = 80%, *α* = 0.05) (Tabachnick & LS Fidell, 2007).

A previous pharmacist survey had been conducted on a random sample of 400 community pharmacies in Surabaya (60% response rate), providing data on the characteristics of the community pharmacies and diabetes services provided (reported elsewhere) (Wibowo et al., 2015). Based on the pharmacy characteristics data, 10 community pharmacies were purposefully selected as sampling sites in this study, aiming to include different geographical areas and socio-economic levels in Surabaya. Surabaya consists of 31 sub-districts which can be categorised into five geographical areas, namely: centre, west, east, north and south; and four socio-economic levels, from high (labelled ‘1’) to low (labelled ‘4’) (Nugroho, 2010). Each pharmacy was responsible for recruiting approximately 20 patients. Patients eligible for the survey were those aged over 18 years, with a diagnosis of type 2 diabetes for which they were receiving oral antidiabetic medications. Patients were recruited as they were seeking oral antidiabetic medications at these pharmacies, and their written consent was obtained.

### Data collection

#### Questionnaire development

The survey questionnaire consisted of four sections: (A) patient demographics, (B) services for type 2 diabetes patients—use of services and views on pharmacists’ roles, (C) diabetes profile, and (D) monitoring profile. The questionnaire cover page contained information about the study and a consent form. Section B of the questionnaire contained a list of services for type 2 diabetes patients that was drafted based on a generic model generated from the literature (Perkumpulan Endokrinologi Indonesia, 2011; Power et al., 2006; Diabetes Australia, 2012; American Diabetes Association, 2013; Department of Health Western Australia, 2008). A binary choice question was used to capture patient usage of each service (‘yes’/’no’), and a 6-point Likert scale was used to reflect patient views on pharmacists’ roles (1 = definitely no, 6 = definitely yes). This was followed by an open-ended question to explore patient priorities regarding their views of pharmacist roles: *‘In your opinion, what are the five most important services that should be provided at pharmacies to assist you with your diabetes?’* The questionnaire was face and content validated by a panel of seven academics, two board members of the IAI, two Indonesian community pharmacists and two diabetes patients. Their feedback, where appropriate, was incorporated into the questionnaire.

The questionnaire (English version) then went through a translation process to an Indonesian version: (i) forward translation to Bahasa Indonesia by one of the investigators whose first language is Bahasa Indonesia; (ii) back-translation to English by an independent English first-language translator; and (iii) the back-translation was compared to the original version by two of the investigators whose first language was English. The forward-translation questionnaire was piloted by 10 type 2 diabetes patients. This resulted in minor changes to the final questionnaire. To assess reliability, the questionnaire was distributed on two occasions separated by a two-week interval. Responses to the Likert scales were grouped (ratings of 1–4, and ratings of 5–6) to ensure that Kappa was able to be calculated; the resulting Kappa scores for diabetes services (Section B) ranged from 0.412 to 1.000, which were classified as ‘acceptable’ to ‘excellent’ levels of test–retest reliability (Landis & Koch, 1977).

#### Questionnaire administration

Owners of the 10 selected pharmacies were approached. In the case of refusal, that pharmacy was replaced with another pharmacy in the same geographical area and socio-economic level (Table 1). At each pharmacy, 40 questionnaires were issued, and the pharmacist and/or pharmacy staff member was briefed about the study and how to complete the questionnaire. The pharmacist and/or the pharmacy staff member was asked to explain about the study to the eligible patients and to invite them to participate; each pharmacy aimed to recruit approximately 20 patients. Once completed, the questionnaire was placed in a sealed envelope by the respondent and submitted to the pharmacist/pharmacy staff. The completed questionnaires as well as the remaining unused questionnaires were then handed to the investigators by the agreed deadline.

**Table 1:** Patient recruitment data from 10 community pharmacies.

Pharmacy code	Geographical area	Socio-economic level	Average number of diabetes patients per month	Number of questionnaires distributed	Number of useable questionnaires returned
Pharmacy 1	East Surabaya	2	150	40	21
Pharmacy 2	Central Surabaya	1	200	28	18
Pharmacy 3	East Surabaya	3	70	35	20
Pharmacy 4	West Surabaya	4	100	32	19
Pharmacy 5	South Surabaya	3	140	37	20
Pharmacy 6	Central Surabaya	2	100	30	20
Pharmacy 7	South Surabaya	4	100	36	20
Pharmacy 8	North Surabaya	1	240	31	19
Pharmacy 9	West Surabaya	3	120	40	20
Pharmacy 10	North Surabaya	2	100	27	19
**Total**				**336**	**196**

### Data analysis

Descriptive statistics were used to summarise the patient characteristics: demographics (Section A), diabetes profile (Section C), and monitoring profile (Section D). SPSS version 19.0 was used to perform the analysis.

In relation to diabetes services (Section B), frequencies were calculated for binary responses (‘yes’/’no’) related to the patient usage of services and for responses from Likert scales related to the extent of patient agreement regarding pharmacists’ roles. Moreover, content analysis was used for responses from the open-ended question to explore patient views on the five priority services that should be provided at the pharmacies. An initial coding frame structure was established from the generic model generated from the literature. The responses were coded (if new codes emerged, they were added to the thematic codes in the coding frame), and frequencies were calculated for each code (Rose, Spinks & Canhoto, 2015).

It was of particular interest to identify characteristics associated with the views of patients related to pharmacy-based diabetes services. For this reason, the responses regarding patients’ views for each type of service were classified into binary variables, to indicate ‘strong agreement’ (Likert scale ratings of 5–6) versus ‘ambivalence or disagreement’ (Likert scale ratings of 1–4) that a service should be provided by the pharmacy. These binary variables were used as dependent variables in logistic regression models to identify patient characteristics associated with the strong support for each role. Some roles were considered to be different aspects of an overarching role. For example, the roles to provide information on: the taking of medications, use of insulin devices, storage of medications, precautions, and adverse effects were all components of ‘medication education.’ In order to analyse characteristics associated with strong support for an overarching or ‘composite’ role, the arithmetic average of the Likert responses for the component roles was calculated and then converted to a binary variable in a manner similar to that used for the individual roles (scores of 5 or more were taken to indicate strong support, otherwise ambivalence or low support). Taking the simple average of the component roles implicitly gives equal weight to each of the roles within a composite. It would have been preferable to use Factor Analysis to identify if some of the component roles were more important than others, but the sample size was considered too small for this refinement. It is generally recommended that sample sizes of 300 or more should be used to obtain stable factor loadings (Tabachnick & LS Fidell, 2007). Patient characteristics included as independent variables were gender, age, education, employment, income, health insurance cover, diabetes organisation membership, duration of diabetes (time since diagnosis), risk factors for complications, complications and diabetes (glycaemic) control. The models also included an independent variable indicating patients’ previous use of the service (binary responses: ‘yes’/’no’). For dependent variables which were ‘composite’ (for example ‘medication education’), the binary responses indicating previous use of each component service were treated as numeric (zero for ‘No’, one for ‘Yes’), and their mean was calculated and used in the model as an independent variable to show the degree of previous use of the composite service. A mean value close to 1 indicated that most components of the composite role had been used, while a lower value indicated less use. A backward elimination strategy was used to identify all the variables which significantly contributed to each model. Through this approach all independent variables were included initially, and then the least significant variable was dropped (one at a time) until the *p*-value associated with each of the variables remaining in the model was less than 0.05.

## Results

### Sample recruitment

This study included 10 community pharmacies as sampling points after approaching 11 community pharmacies in Surabaya. One pharmacy refused, as the employee pharmacist was planning to resign and they were in the process of recruiting a new one. From the final 10 pharmacies, a total of 336 questionnaires were distributed and 204 were returned; however, eight patients reported the use of insulin at the beginning of their therapies and were deemed to have type 1 diabetes, leaving a sample size of 196 (a response rate of 58.3%) (Table 1).

### Characteristics of pharmacy patients with type 2 diabetes

The demographic information of participating patients (Section A) is summarised in Table 2. Approximately 60% of respondents were female and half of respondents were aged 60 years or older. Approximately half of the respondents did not have health insurance plans.

**Table 2:** Demographic data of patient respondents (*N* = 196).

Patient demographics	Frequency (%)
*Gender*	
Male	80 (40.8)
Female	116 (59.2)
*Age, years*—*median (range)*	60.0 (32–86)
*Ethnicity*	
Asian	196 (100.0)
Others	0 (0.0)
*Highest education*	
No schooling	6 (3.1)
Primary school	23 (11.7)
Junior high school	41 (20.9)
Senior high school	71 (36.2)
Diploma	18 (9.2)
Bachelor degree	25 (12.8)
Postgraduate degree	12 (6.1)
*Employment status*	
Working full-time (≥40 hours/week)	53 (27.0)
Working part-time (<40 hours/week)	20 (10.2)
Not working	123 (62.8)
*Total household income (from all sources) per month* ^a^	
≤Rp 2 million	103 (52.6)
>Rp 2 million—5 million	54 (27.6)
>Rp 5 million—10 million	25 (12.8)
>Rp 10 million	12 (6.1)
*Health insurance*	
Self-sponsored insurance	31 (15.8)
Employer-sponsored insurance	56 (28.6)
Insurance scheme for the poor/near poor	9 (4.6)
No insurance	100 (51.0)
*Member of a diabetes organisation*	
Yes	74 (37.8)
No	122 (62.2)

**Notes.**

Abbreviations Rp Indonesian rupiah

a 2 missing responses.

The diabetes data reported by participating patients (Section C) are shown in Table 3. The median duration of diabetes (time since diagnosis) was seven years. More than 80% of respondents reported that their treatment regimen included oral antidiabetic medications and diet modifications. It is important to note that this variable did not measure whether the doctor made the medication/diet/exercise recommendations, but rather whether the patient remembered and/or reported it. Almost 60% of the respondents reported having at least one diabetes-related complication, and most of the patients reported having at least one risk factor for complications.

**Table 3:** Self-reported diabetes and health profile of patient respondents (*N* = 196).

Patient diabetes profile	Frequency (%) of ‘yes’
*Duration of diabetes, years—median (range)* ^a^	7 (1–42)
*Current diabetes treatment*	
Modifying diet	173 (88.3)
Exercise programme	123 (62.8)
Oral antidiabetic medication	189 (96.4)
Insulin	44 (22.4)
*Risk factors* ^c^	157 (80.1)
BMI ≥25 kg/m^2^^b^	88 (44.9)
(History of) smoking	41 (20.9)
High cholesterol^d^	77 (39.5)
High blood pressure^e^	104 (53.6)
*Complications* ^c^	115 (58.7)
Heart disease	34 (17.3)
Eye problems	52 (26.5)
Foot discomfort	81 (41.3)
Foot ulcers	14 (7.1)
Kidney problems	16 (8.2)

**Notes.**

a Some missing responses.

b BMI, body mass index = weight (kg) divided by height^2^ (m^2^); some missing responses.

c Respondents responded ‘yes’ for at least one complication/risk factor.

d Respondents responded ‘yes’, either for *“Do you have high cholesterol?” or “Do you take medications to treat your high cholesterol?”*, or for both.

e Respondents responded ‘yes’, either for *“Do you have high blood pressure?” or “Do you take medications to treat your high blood pressure?”,* or for both.

Table 4 shows the monitoring profile reported by participating patients (Section D). HbA1c refers to glycosylated hemoglobin, which identifies average plasma glucose concentration over the previous three months. To provide insight to patients’ diabetes (glycaemic) control, variables related to the symptoms of hypo/hyperglycaemia and HbA1c values were combined to indicate: fair-good control (i.e., no symptoms and HbA1c ≤8.0%); poor control (i.e., presence of symptoms and/or HbA1c >8.0%); and unknown (i.e., symptoms ‘none/don’t know’ and/or HbA1c values ‘none/don’t know’). Using this derived variable, most respondents were perceived to have either poor diabetes control (45.9%) or unknown diabetes control (42.3%).

**Table 4:** Self-reported monitoring profile of patient respondents (*N* = 195).^a^

Patient monitoring	Frequency (%) of ‘yes’
**Diabetes (glycaemic) control**	
*High blood sugar reactions (in the last month)*	56 (28.7)
*Low blood sugar reactions (in the last month)*	43 (22.1)
*Severe blood sugar reactions (in the last year)*	26 (13.3)
*HbA1c last value*	53 (27.0)^b^
<6.5%	18
6.5–8%	27
>8%	8
**Routine tests**	
*SMBG (in the last week)*	74 (37.9)
*Medical monitoring (in the last 3 months)*	
Blood sugar	167 (86.1)
Blood pressure	166 (85.1)
Weight	124 (63.9)
*HbA1c measurement (in the last year)*	65 (33.3)
*Medical monitoring (in the last year)*	
Cholesterol	127 (65.1)
Kidney	84 (43.1)
Eyes	48 (23.2)
Feet	41 (21.0)

**Notes.**

Abbreviations: SMBG self-monitoring of blood glucose HbA1c glycosylated haemoglobin

a 1 missing response.

b Number of respondents reported their HbA1c last value.

### Patients’ use of pharmacy services and their views on pharmacist roles

Responses describing patient usage of pharmacy services are summarised in Table 5. In addition to the traditional role of dispensing (‘treatment administration’), the most frequent services received were ‘patient education’ about medications, particularly directions for use (79.6%) and special precautions to follow (71.9%).

**Table 5:** Patients’ use of community pharmacy services and their views on pharmacists’ roles (*N* = 196).

Services	Being used *N* (%)	Being viewed as pharmacist roles^a^*N* (%)
**Treatment administration**		
Prepare medications	196 (100)	195 (100)
Provide labels with instructions for use	196 (100)	195 (100)
**Patient education**		
Disease process	93 (47.4)	120 (61.5)
Treatment targets	79 (40.3)	115 (59.0)
Antidiabetic medications:		
Directions for use	156 (79.6)	160 (82.1)
Use of insulin devices^b^	27 (61.4)	142 (72.7)
Storage requirements	93 (47.4)	144 (73.9)
Special precautions to follow	141 (71.9)	155 (79.5)
Common/important adverse effects	87 (44.4)	139 (71.3)
Exercise	66 (33.7)	96 (49.2)
Diet	84 (42.9)	101 (51.8)
SMBG	63 (32.1)	107 (54.9)
Prevention/treatment of acute complications	67 (34.2)	126 (64.6)
Prevention/treatment of chronic complications	45 (23.0)	116 (59.5)
Need for regular medical monitoring	48 (24.5)	97 (49.8)
Foot self-care	35 (17.9)	95 (48.7)
Smoking cessation^c^	12 (29.3)	72 (36.7)
**Monitoring**		
Monitor compliance with:		
Antidiabetic medications	100 (51.0)	127 (65.1)
Exercise plan	62 (31.6)	102 (52.3)
Diet plan	78 (39.8)	109 (55.9)
Plan for prevention/treatment of chronic complications	44 (22.4)	92 (47.2)
Scheduled medical monitoring	38 (19.4)	96 (49.3)
Monitor treatment outcomes:		
Check records on SMBG	58 (29.6)	101 (51.8)
Carry out blood glucose tests	58 (29.6)	113 (58.0)
Measure BMI	40 (20.4)	89 (45.6)
Measure blood pressure	55 (28.1)	103 (52.8)
Check results on patient laboratory tests	51 (26.0)	98 (50.3)
Monitor for adverse effects	63 (32.1)	110 (56.4)
**Review**		
Refer patients if necessary	69 (35.2)	110 (56.5)

**Notes.**

Abbreviations SMBG self-monitoring of blood glucose BMI body mass index

a 1 missing response.

b The percentage was calculated for patients currently/previously taking insulin (*N* = 44).

c The percentage was calculated for patients currently (or had a history of) smoking (*N* = 41).

Table 5 also shows responses regarding pharmacists’ roles. All patients agreed with pharmacists’ roles in dispensing. Beyond dispensing, more than 70% of respondents expected pharmacists to provide ‘patient education’ about medications. About half of respondents supported other activities related to ‘patient education’ and ‘monitoring’. There were large gaps between patient usage and expectation regards education on: medication storage and adverse effects, SMBG, prevention of complications, need for regular monitoring, and foot care. In addition, patients expected pharmacists to provide more monitoring services than those currently provided.

In terms of the priority roles of pharmacists, patients’ responses can be seen in Table 6. The top five services perceived by patients as priorities (in addition to pharmacists’ traditional roles of dispensing being already provided) were from the ‘patient education’ domain—education related to medications (i.e., directions for use (64.5%), common/important adverse effects (25.5%), storage requirements (26.6%)) and the ‘monitoring’ domain—monitoring compliance with medications (37.3%). No new services were raised, beyond those already listed in the questionnaire, from this open-ended question, suggesting that from the respondents’ perspective the range of services that should be provided by community pharmacies described in Table 5 was complete.

**Table 6:** Patients’ open-ended views on priority roles of pharmacists in diabetes care (*N* = 169).^a^

Priority services^b^	Number of responses (%)
**Treatment administration**	
Prepare medications	35 (20.7)
Provide labels with instructions for use	66 (39.1)
**Patient education**	
Disease process	27 (16.0)
Antidiabetic medications:	
Directions for use	109 (64.5)
Use of insulin devices	15 (8.9)
Storage requirements	45 (26.6)
Special precautions to follow	37 (18.9)
Common/important adverse effects	50 (25.5)
Exercise	27 (13.8)
Diet	33 (16.8)
Prevention/treatment of acute complications	27 (16.0)
Prevention/treatment of chronic complications	20 (11.8)
**Monitoring**	
Monitor compliance with:	
Antidiabetic medications	63 (37.3)
Monitor treatment outcomes:	
Carry out blood glucose tests	30 (17.8)
Measure blood pressure	25 (14.8)
Monitor for adverse effects	23 (13.6)
**Others** *(not a specific service)*	
Provide a complete range of medications	12 (7.1)
Information about medications	16 (9.5)

**Notes.**

Responses to an open-ended question:*“In your opinion, what are the five most important services that should be provided at pharmacies to assist you with your diabetes?”*

a From a total 196 respondents, there were 26 missing responses and 1 invalid response, giving a total *N* = 169.

b Services selected by more than 10 respondents.

### Characteristics associated with patients’ views on pharmacist roles

Logistic regression models were used to identify patient characteristics which were associated with patients’ views on pharmacists’ roles. The odds ratios of significant characteristics are summarised in Table 7.

**Table 7:** Odds ratios and 95% confidence intervals of significant characteristics associated with support for ‘patient education’ and ‘monitoring’ by pharmacists.

		Patient education by pharmacists				Monitoring by pharmacists			
	*N*	Medications^a^	Exercise	Diet	All education^b^	Compliance	Treatment outcomes		Adverse drug reaction
							Perform clinical testings^d^	Check test results^e^	
*Income*									
Low	105	Reference	Reference	Reference	Reference	Reference			Reference
Moderate	54	NS	0.4 (0.17–0.90)	NS	NS	NS			NS
High	37	0.3 (0.10–0.72)	0.3 (0.10–0.80)	0.3 (0.12–0.61)	0.3 (0.10–0.68)	0.3 (0.10–0.72)			0.2 (0.09–0.53)
*Employment*									
Not working	123		Reference		Reference	Reference	Reference	Reference	
Working	73		0.3 (0.15–0.83)		0.5 (0.24–0.94)	0.4 (0.21–0.88)	0.5 (0.24–0.97)	0.3 (0.15–0.74)	
*Risk factors*									
No	39								Reference
Yes	157								3.4 (1.46–8.03)
*Diabetes (glycaemic) control* ^f^									
Good/fair	23						Reference	Reference	Reference
Poor	90						NS	4.9 (1.20–20.55)	3.2 (1.05–9.97)
Unknown	83						2.3 (1.22–4.51)	10.2 (2.44–42.95)	4.3 (1.36–13.57)
*Previous use of the service*									
No	^g^	Reference	Reference	Reference	Reference	Reference	Reference	Reference	Reference
Yes		4.5 (1.79–11.53)	10.3 (4.6–23.15)	4.4 (2.30–8.30)	4.5 (1.60–12.51)	5.2 (1.79–11.52)	13.6 (5.21–35.51)	11.3 (4.51–28.13)	6.3 (2.82–13.90)

**Notes.**

a A composite variable—education related to antidiabetic medications: directions for use, use of insulin devices (calculated only from those currently/previously taking insulin), storage, special precautions and common/important adverse effects; a mean rating ≥5 was used.

b A composite variable—all education: disease process, treatment targets, antidiabetic medications, exercise, diet, self-monitoring of blood glucose, prevention/treatment of acute complications, prevention/treatment of chronic complications, need for regular monitoring, foot self-care and smoking cessation (calculated only from those currently, or had a history of, smoking); a mean rating ≥5 was used.

c A composite variable—monitoring compliance with: antidiabetic medications, exercise and diet plan, plan for prevention/treatment of complications and scheduled medical monitoring; a mean rating ≥5 was used.

d A composite variable—perform clinical testings (measuring blood glucose, blood pressure and BMI); a mean rating ≥5 was used.

e A composite variable – check test results (patient self-monitoring records and laboratory data); a mean rating ≥5 was used.

f Diabetes (glycaemic) control is a composite variable of hyper/hypoglycaemia symptoms and HbA1c values.

g Numbers differ for each endpoint (service).

NS not significantly different from the reference

Patient experience (previous use) with a service was strongly associated with their views that the service should be provided by pharmacists (Odds Ratios (ORs) 4.4–11.3). Patients with poor/unknown glycaemic control or those who had risk factors for complications were more supportive of pharmacists providing some monitoring services (ORs 2.3–10.2). On the other hand, patients with higher incomes or those who were working were less supportive towards pharmacists providing some education and monitoring services (ORs 0.2–0.5).

## Discussion

This study has found most type 2 diabetes patients recruited had complications and/or risk factors for complications (80.1% and 58.7%, respectively), and/or had poor/unknown glycaemic control (45.9% and 42.3%, respectively). It is evident that in the current hospital outpatient/clinic treatment model many patients were poorly monitored. This is consistent with a population study of type 2 diabetes patients in Indonesia which reported that 67.9% of type 2 diabetes patients had not achieved good glycaemic control (HbA1c <7.0%), and approximately 60% of the patients had complications and/or risk factors for complications (i.e., dyslipidaemia and/or hypertension) (Soewondo et al., 2010).

Moreover, this study reported that follow-up care tended to be inadequate, with only about one-third of respondents reporting annual HbA1c monitoring and eye or foot examinations. Supporting this finding, the Patient and Health Provider Survey in Indonesia (2012) indicated that the majority of patients had not received foot or eye examinations within the past year, only 30% had had their HbA1c checked and many had expressed a wish to see health care providers more often (Novo Nordisk, 2013). It was suggested that this poor quality of care and patient outcomes might relate to the lack of awareness of, accessibility to and affordability of diabetes care for this patient group (Novo Nordisk, 2013). Together with the findings of this study, it provides a basis for community pharmacists to provide a range of services.

### Patients’ use of pharmacy services and their views on pharmacist roles

This study indicated little involvement of Indonesian community pharmacies in the care of patients with type 2 diabetes. Respondents mostly utilised pharmacists for their supply role (dispensing). A previous Indonesian study has confirmed the limited services provided to general pharmacy patients (Handayani & Raharni, Gitawati, 2009).

Amongst non-supply roles, many respondents chose roles closely related to dispensing as the priority roles of pharmacists, i.e., education related to medications, and monitoring compliance with medications. Similar findings were evident from some international studies involved diabetes patients (Abduelkarem et al., 2003a; Weitzman et al., 2009; Brown & Green, 2000; Hermansen-Kobulnicky & Worley, 2008; Twigg et al., 2013). Two qualitative studies indicated that patients identified the primary expertise of the community pharmacist as medicines supply, and there were mixed perceptions of community pharmacists’ roles extending to advising on prescription medicines, providing disease-related/health advice or providing monitoring services (using clinical testing devices) (Twigg et al., 2013; Gidman & Cowley, 2013).

It is interesting that the responses to the open-ended question that patients perceived that services related to dispensing and patient education were those that should be provided to type 2 diabetes patients from community pharmacies (Table 6). All of those they considered should be provided had been included in the options that could be provided (Table 5). This also indicated that patients did not have additional requirements for services not identified initially for this study.

### Characteristics associated with patients’ views on pharmacist roles

The logistic regression models consistently found that a patient’s support for a service was influenced by their experience (previous use) of the service (Odds Ratios, ORs ≥4.4). Supporting this finding, studies worldwide have shown that type 2 diabetes patients have increased perceptions of pharmacists’ ability to assist them after receiving pharmacy-based services (Fera et al., 2008; Garrett & Martin, 2003; Hughes, 2006; Abduelkarem & Sackville, 2009; Hales et al., 2010). It should be emphasised that most patients in this study (at that time) received limited services from community pharmacies, thus they might not be aware of what pharmacists should and could do.

The implementation of *Jaminan Kesehatan Nasional*—JKN (National Health Coverage) in 2014 provides the best opportunity to optimise the use of Indonesian community pharmacies. It is important for the Government and IAI to establish an agreement on the basic services that should be available in community pharmacies. While the current payment under the scheme includes a very low prescription fee (Kementrian Kesehatan Republik Indonesia, 2014), the IAI should negotiate adequate remuneration for pharmacists to provide the services, thus enabling community pharmacies to remain viable.

In addition to patients’ past experiences, patients who were working and/or had higher incomes were generally less supportive of some of the proposed education or monitoring services (ORs ≤0.5). This might be because these groups of patients were likely to be younger (mean age 63.7 years for non-workers versus 54.0 years for workers, *p* < 0.0001; and mean age 60.4 years for income ≤Rp 5 million versus 58.6 years for income >Rp 5 million, *p* = 0.358). Two previous studies have reported that older patients, or those living with diabetes for a long time, were more supportive of pharmacists’ contributions (Twigg et al., 2013; Ibrahim, Al Tukmagi & Wayyes, 2013). It has been suggested that elderly people are one of the groups whose need for additional advice on medications and other related services has been demonstrated (Cartwright & Smith, 1988).

Notably, patients who had risk factors for complications and/or had poor/unknown glycaemic control were much more supportive of pharmacists monitoring treatment outcomes or adverse drug reactions (ORs ≥2.3). Such patients might reflect those with lower health status, representing a target group who might be more motivated and responsive to pharmacists’ involvement. It has been suggested that patients who benefit most from pharmacist-led education/coaching and disease state management services include those with poor glycaemic control and multiple comorbidities (Sisson & Kuhn, 2009). It is evident that overall treatment outcomes in this patient cohort are concerning and expanding the community pharmacy role into patient management needs to be investigated.

### Limitations

The purposeful sampling method used in the study (20 patients from each of 10 pharmacies) was considered the only feasible manner by which it could be conducted. The request for approximately 20 patients was to ensure that each pharmacy included a range of patients in the sample and in many cases this about half of their current type 2 diabetes patients. No full list (sampling frame) of pharmacy patients with diabetes exists in Surabaya, so it was not possible to obtain a truly random sample of patients with this condition. Thus, there is a possibility of non-respondents not sharing the same practice and/or views of respondents, and some caution should be exercised in generalising the findings. However, the pharmacies covered a wide range of settings (geographic and socioeconomic), so that no particular background group of patients would be excluded; and achieved a sound response rate of approximately 60%. The characteristics of the respondents in this study were comparable to those of a population study involving all type 2 diabetes patients visiting 18 medical centres across Indonesia between November 2008 and February 2009 (*N* = 1,785) with respect to age (60 years *versus* 59 years, respectively), gender (female 59.2% *versus* 55.2%, respectively), and duration of diabetes (7 years *versus* 8 years, respectively) (Soewondo et al., 2010). Hence, although the sample is not randomly selected and the risk of response bias might limit the generalisation, the views of participants give some insight into the diabetes services used or desired at community pharmacies in Surabaya.

## Conclusions

Community pharmacies in Surabaya, Indonesia in this study are mainly utilised for their basic services of dispensing. Many type 2 diabetes patients in these pharmacies reported limited monitoring of blood glucose and poor glycaemic control; in addition, their follow-up care and health outcomes were generally poor. These findings indicate a need and opportunities for community pharmacists to provide a range of services for patients with diabetes. Hence, strategies should be developed to broaden current pharmacy patients’ limited views of pharmacists’ roles which are mainly perceived as extensions to the supply roles. It is evident that patients support the provision of services once they have been provided. Patient characteristics that influence these views provide a target group for implementation of a pharmacy-based diabetes service that should be evaluated. This can provide a partial solution in the environment of a burgeoning burden of diabetes in Indonesia.

## Supplemental Information

10.7717/peerj.1449/supp-1 Supplemental Information 1 Responses to patient questionnaire

## Additional Information and Declarations

### Data Availability

The following information was supplied regarding data : Raw data can be found in the Supplemental Information.
